# Latest Developments in “Adaptive Enrichment” Clinical Trial Designs in Oncology

**DOI:** 10.1007/s43441-024-00698-3

**Published:** 2024-09-13

**Authors:** Yue Tu, Lindsay A. Renfro

**Affiliations:** Division of Biostatistics, Department of Population and Public Health Sciences, University of Southern California, Los Angeles, CA USA

**Keywords:** Biomarker-driven trials, Adaptive enrichment, Trial design, Cancer clinical trials

## Abstract

As cancer has become better understood on the molecular level with the evolution of gene sequencing techniques, considerations for individualized therapy using predictive biomarkers (those associated with a treatment’s effect) have shifted to a new level. In the last decade or so, randomized “adaptive enrichment” clinical trials have become increasingly utilized to strike a balance between enrolling all patients with a given tumor type, versus enrolling only a subpopulation whose tumors are defined by a potential predictive biomarker related to the mechanism of action of the experimental therapy. In this review article, we review recent innovative design extensions and adaptations to adaptive enrichment designs proposed during the last few years in the clinical trial methodology literature, both from Bayesian and frequentist perspectives.

## Introduction

As cancer has become better understood on the molecular level with the evolution of gene sequencing techniques, considerations for individualized therapy using predictive biomarkers (those associated with a treatment’s effect) have shifted to a new level. Traditional randomized trial designs tend to either oversimplify or overlook differences in patients’ genetic and molecular profiles, either by fully enriching eligibility to a marker subgroup or enrolling all-comers without prospective use of potentially predictive biomarkers. In the former case of marker enrichment, one cannot learn about a marker’s true predictive ability from the trial’s conduct (as marker-negative patients are excluded); in the latter case ignoring the biomarker, the end result may be a “washing out” of the treatment effect when a predictive marker truly does exist within the sampled patient population.

In the last decade or so, randomized “adaptive enrichment” clinical trials have become increasingly utilized to strike a balance between enrolling all patients with a given tumor type, versus enrolling only a subpopulation whose tumors are defined by a potential predictive biomarker related to the mechanism of action of the experimental therapy (see for example [1, 2]). On a high level, adaptive enrichment designs take the form of a clinical trial that begins by randomizing participants to a targeted versus a control therapy regardless of marker value, then adapts through a series of one or more interim analyses to potentially limit subsequent trial recruitment to a marker-defined patient subpopulation that is showing early signals of enhanced treatment benefit.

In this review article, we first discuss the “traditional” presentation of both enrichment and adaptive enrichment designs and their decision rules and describe statistical or practical challenges associated with each. Next, we introduce innovative design extensions and adaptations to adaptive enrichment designs proposed during the last few years in the clinical trial methodology literature, both from Bayesian and frequentist perspectives. Finally, we review articles in which different designs within this class are directly compared or features are examined, and we conclude with some comments on future research directions.

### Enrichment Trial Designs

To motivate discussion of adaptive enrichment designs and why they are useful, it is helpful to first understand *enrichment trial designs*, or designs that focus only on a subset of the patient population from the beginning.

**Figure 1.**
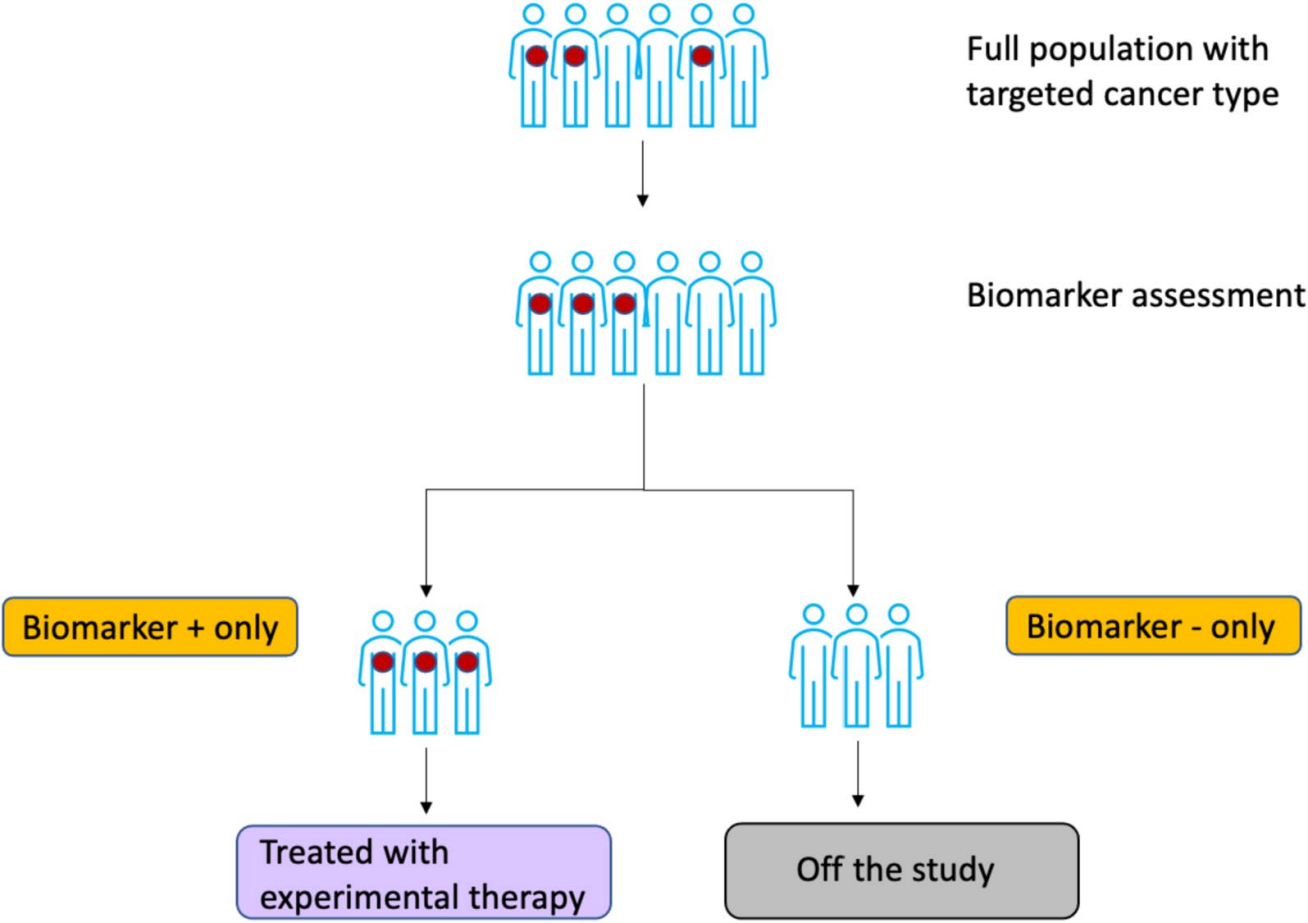
Enrichment trial Schema with a Single Arm.

Design Details: In the setting of targeted therapies with strong prior evidence or clinical rationale supporting efficacy only within a biomarker-selected subgroup, “marker-enriched” or enrichment trial designs are used to confirm signal or efficacy only in that selected subgroup. In these types of trials, patients are screened and classified into prespecified marker positive and negative subgroups at or prior to enrollment, with only marker positive patients eligible to remain on study and receive protocol-directed targeted therapy. This usually takes the form of a small, single-arm phase II study without a randomized comparator, but in some settings, comparisons against a randomized non-targeted standard of care therapy might be made (see Fig. 1).

Example: An example of a clinical trial with an enrichment design is the Herceptin Adjuvant (HERA) trial. The HERA trial is a phase III, randomized, three-arm trial that studied the efficacy of 1 year versus 2 years of adjuvant trastuzumab versus control (no additional treatment) in women with human epidermal growth factor receptor 2 (HER2)-positive early breast cancer after completion of locoregional therapy and chemotherapy [3]. HER2 is overexpressed in 15–25% of breast cancer and trastuzumab, a monoclonal antibody, binds the HER2 extracellular receptor [4, 5]. The primary outcome was disease-free survival and using an intention-to-treat analysis, significant treatment benefit was demonstrated for 1 year of trastuzumab compared to the control arm.

Limitations: One important limitation of enrichment designs is that a marker’s predictive ability to select patients for treatment is assumed to already be known and cannot be validated from the trial itself. It is theoretically possible that a pre-defined marker-negative subgroup might also benefit from the targeted treatment, but that knowledge won’t be updated with an enrichment design. For example, a pre-clinical study found that trastuzumab can decrease cancer cell proliferation in HER2 negative and HER2 phosphorylation at tyrosine Y877 positive breast cancer cell lines, which is comparable to the drug effect in HER2 positive breast cancer cell lines, showing that the HER2 negative subpopulation may also benefit from trastuzumab [6]. Around the same time, however, the randomized study B-47 conducted by the National Surgical Adjuvant Breast and Bowel Project (NSABP) group showed no effect of trastuzumab in HER2-low patients [7].

Another limitation of enrichment trial designs is the necessity of establishing predefined subgroups during the study planning phase, which becomes complicated when dealing with biomarkers that are measured on a continuous scale, like expression levels or laboratory values. Determining an appropriate threshold to divide patients into “positive” and “negative” groups is not always straightforward, validated, or effective in distinguishing the effect of the targeted treatment. Selecting an incorrect threshold during trial design can result in an ineffective or underpowered study, and revising the decision once the trial has begun accrual is not advisable.

### Adaptive Enrichment Trial Designs

*Adaptive enrichment* trial designs, on the other hand, are an attractive solution to the inherent weaknesses of a fully enriched trial design.

**Figure 2.**
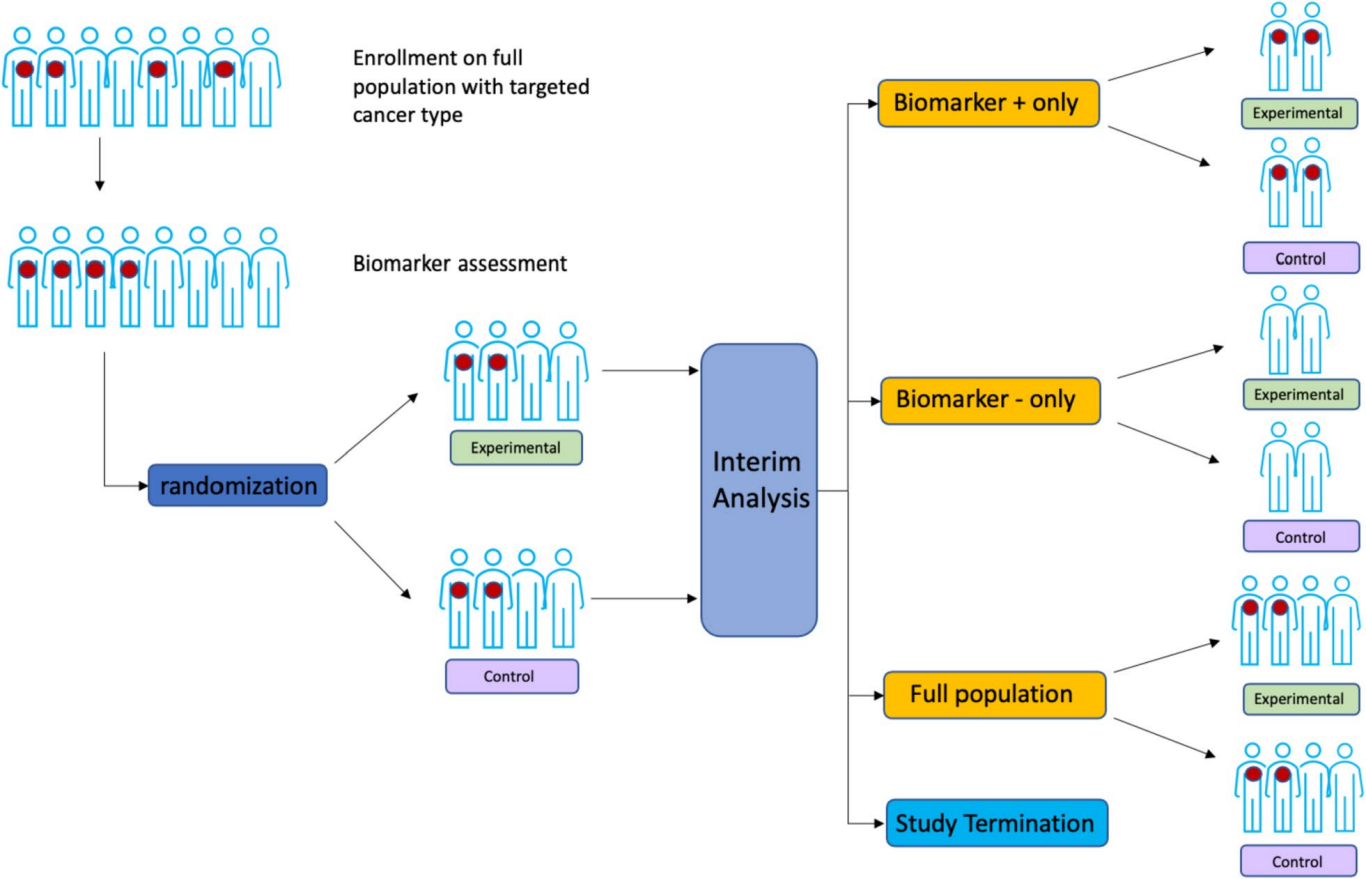
Adaptive Enrichment Trial Schema with a Binary Biomarker.

Design Details: An adaptive enrichment trial design initially enrolls patients with any marker value(s) and randomizes them to experimental targeted versus standard (non-targeted) therapy. As the trial progresses, accrual may be subsequently refined or restricted to patients with certain marker values according to those showing initial efficacy on the basis of one or more interim analyses. This design is randomized out of necessity, so that treatment-by-marker interactions may be computed, and adaptations based on differential treatment effects by marker subgroups can be facilitated. At the interim analyses, according to pre-specified decision rules, a trial may stop early for futility or efficacy, either overall, or within a marker-defined subgroup. If the biomarker of interest is not naturally dichotomous, the same interim analyses may also be used to select or revise marker cutpoints (see Fig. 2).

Example: One real-world example of an adaptive enrichment design is the Morphotek Investigation in Colorectal Cancer: Research of MORAb-004 (MICRO), which is an adaptive, two-stage, phase II study assessing the effect of ontuxizumab versus placebo in patients with advanced metastatic colorectal cancer [8, 9]. Ontuxizumab, a monoclonal antibody treatment targeting endosialin function, was expected to be more effective in patients with endosialin-related biomarkers. Since the biomarkers were continuous in nature and the optimal cutoffs were unknown, the study included an assessment for determining the best cutoffs at an interim analysis, where progression-free survival (PFS) served as the primary endpoint. Initially, the goal was to demonstrate the treatment effect of ontuxizumab either overall or within subgroups defined by biomarkers. However, the interim analysis revealed that none of the biomarkers had a predictive relationship with treatment outcome. Consequently, the design shifted to a non-marker-driven comparison. Additionally, the interim analysis showed early futility for ontuxizumab compared to placebo overall, terminating the trial early due to lack of efficacy. In summary, this adaptive enrichment design concluded both the biomarker assessment and the evaluation of the therapy early, and additional resources and patients were spared. However, it is worth noting that it may have been underpowered to identify modestly-sized interaction effects, had they been present.

Limitations: Adaptive enrichment trial designs do have some statistical challenges, including limitations faced in the design of the MICRO trial. These include estimation of subgroup-specific treatment effects, particularly when the marker prevalence is low, as a sufficiently large sample size is required to have enough patient-level information at interim analysis for informative subgroup selection. As a practical consideration, the primary endpoint must be quickly observed relative to the pace of accrual, to allow time for impactful adaptations based on observed outcomes relatively early in the trial. Another challenge is how exactly one should select cutpoints for adaptation of accrual. In the MICRO trial, at the interim analysis, a series of Cox proportional hazards models were fit over a grid of possible cutpoints, and the significance of a marker by treatment interaction term was evaluated. A pre-specified level of statistical significance for the interaction, along with a clinically meaningful effect in the marker “positive” group defined by the interaction, would warrant potential accrual restriction; however, this approach treated truly continuous biomarkers as binary in its implementation, which results (at least theoretically) in a loss of information and potential loss of power.

Several groups have attempted to extend or modify the standard adaptive enrichment trial design in various ways to address statistical shortcomings or tailor the strategy to various applications. The remainder of this paper provides an overview of some of these recent developments. While we admit such designations are rather arbitrary, we present this work separately by Bayesian and frequentist approaches, so that structural similarities among them may be readily described and compared.

## Recent Developments and Extensions in Adaptive Enrichment Trial Designs

### Bayesian Approaches

Xu et al. proposed an adaptive enrichment randomized two-arm design that combines exploration of treatment benefit subgroups and estimation of subgroup-specific effects in the context of a multilevel target product profile, where both minimal and targeted treatment effect thresholds are investigated [10]. This adaptive subgroup-identification enrichment design (ASIED) opens for all-comers first, and subgroups identified as having enhanced treatment effects are selected at an interim analysis, where pre-set minimum and targeted treatment effects are evaluated against a set of decision criteria for futility or efficacy stopping for all-comers or possible subgroups. A Bayesian random partition (BayRP) model for subgroup-identification is incorporated into ASIED, based on models proposed by Xu et al. and Guo et al. [11, 12]. Due to the flexibility of the BayRP model, biomarkers can be continuous, binary, categorical, or ordinal, and the primary endpoint types can be binary, categorical, or continuous. Per the authors, extensions to count or survival outcomes are also possible. BayRP was implemented due to its robustness, but other Bayesian subgroup identification methods could be used as well, like Bayesian additive regression tree (BART) or random forests for larger sample sizes [13]. A tree-type random partition of biomarkers is used as a prior and an equally spaced k-dimensional grid constructed from k biomarkers is used to represent possible biomarker profiles. The operating characteristics of ASIED as a trial design was evaluated by simulations with 4 continuous biomarkers, a total sample size of 180, an interim analysis after 100 patients were enrolled, a minimum desired treatment effect of 2.37 and target treatment effect of 3.08 on a continuous score scale. ASIED’s recommendations were close to the expected results. However, the number of simulated trials was only 100, which could yield lower precision of the estimated operating characteristics. Another limitation is that the partition of the biomarker profile was limited to at most four biomarker subgroups due to the small sample size in each partition.

Another Bayesian randomized group-sequential adaptive enrichment two-arm design incorporating multiple baseline biomarkers was proposed by Park et al. [14]. The design’s primary endpoint is time-to-event, while a binary early response acts as a surrogate endpoint assisting with biomarker pruning and enrichment to a sensitive population at each interim analysis. Initially, the study is open for all-comers and the baseline biomarkers can be binary, continuous, or categorical. The first step at each interim analysis is to jointly select covariates based on both the surrogate and final endpoints by checking each treatment by covariate interaction. The second step is to recalculate the personalized benefit index (PBI), which is a weighted average posterior probability indicating patients with selected biomarkers who benefit more from the experimental treatment. The refitted regression from the variable selection step will redefine the treatment-sensitive patients, and only patients with PBI values larger than some pre-specified cutoff continue to be enrolled to the trial. The third step is to test for futility and efficacy stopping by a Bayesian group sequential test procedure for the previously identified treatment-sensitive subgroups. In simulations, AED was compared with group sequential enriched designs called InterAdapt and GSED, an adaptive enrichment design and all- comers group sequential design [15–17]. The maximum sample size considered was 400, and patients were accrued by a Poisson process with 100 patients per year. Two interim analyses took place after 200 and 300 patients enrolled, and 10 baseline biomarkers were considered. Across each of the seven scenarios, prevalence of the treatment-sensitive group was set to be 0.65, 0.50, or 0.35. While nearly all the designs controlled the nominal Type I error to 0.05, AED had higher probabilities of identifying the sensitive subgroup and correctly concluding efficacy than other designs. Also, 1000 future patients were simulated and treated by each design’s suggested treatment, and AED had the longest median survival time overall. One stated limitation of this work was its inability to handle high dimensional baseline biomarker covariates, as the authors suggest considering no more than 50 baseline covariates in total. Also, biomarkers in this design are assumed to be independent, though selection adjustment for correlated predictors is mentioned. It is worth noting that early response (as used by this design) has not been validated as a good surrogate for longer-term clinical endpoints.

To address the scenario of a single continuous predictive biomarker where the marker-treatment relationship is continuous instead of a step function, Ohwada and Morita proposed a Bayesian adaptive patient enrollment restriction (BAPER) design that can restrict the subsequent enrollment of treatment insensitive biomarker-based subgroups based on interim analyses [18]. The primary endpoint is assumed to be time-to-event, and the relationship between the biomarker and treatment effect is assumed to increase monotonically and is modeled via a four-parameter change-point model within a proportional hazard model. Parameters are assumed to follow non-informative priors, and the posterior distributions are calculated using the partial likelihood of the Cox proportional hazard model. At each interim analysis, decisions can be made for a subgroup or the overall cohort. In addition, treatment-sensitive patients can be selected based on a biomarker cutoff value, which is determined by searching over the range of biomarker values and picking the one with the highest conditional posterior probability of achieving the target treatment effect. Simulations were conducted to compare the proposed method against both a similar method without enrichment and a design using a step-function to model marker-treatment interaction effects without enrichment. The maximum sample size considered was 240 with two interim analyses, and the assumed target hazard ratio was 0.6. The results show that the proposed BAPER method decreases the average number of enrolled patients who will not experience the targeted treatment effect, compared to designs without patient selection. Also, BAPER has a higher probability of correctly identifying the cutoff point that achieves the target hazard ratio. However, BAPER has certain restrictions: the biomarker cannot be prognostic, as the main effect for the biomarker is excluded from the proportional hazard model. Also, the design does not consider the distribution of the biomarker values themselves, so a larger sample size is required when the prevalence of the treatment sensitive (or insensitive) population is small.

Focusing on an optimal decision threshold for a binary biomarker which is either potentially predictive or both prognostic and predictive, Krisam and Kieser proposed a new class of interim decision rules for a two-stage, two-arm adaptive enrichment design [19]. This approach is an extension of Jenkins et al.’s design but with a binary endpoint instead of a time-to-event outcome [20]. Initially, their trial randomizes all patients from two distinct subgroups (i.e., a binary biomarker), assuming one subgroup will have greater benefit, and the sample size is fixed per stages by treatment group. At the first interim analysis, the trial might stop early for futility, continue enrolling to only the marker-positive group, or continue enrolling the full population, while using Hochberg multiplicity- corrected p-values for these decisions. When the full population proceeds to the second stage, it remains possible that efficacy testing will be performed both overall and in the treatment-sensitive subgroup if the biomarker is found to be predictive or prognostic, or only within the total population if the biomarker is not predictive. The critical boundaries for subgroup decisions minimize the Bayes risk of a quadratic loss function by setting the roots of partial derivatives as optimal thresholds, assuming the estimated treatment effects follow bivariate normal distributions with design parameters from uniform prior distributions. A relevance threshold for the effect size, which serves as the minimal clinical meaningful effect, also needs to be prespecified. Optimal decision threshold tables are presented for a biomarker that is predictive, both predictive and prognostic, or non-informative, with sample sizes ranging from 20 to 400 and subgroup prevalence values of 0.1, 0.25 and 0.5 considered. In their simulations, the sample size is 200 per group per stage (for a total trial sample size of 800), the treatment effect (response rate) in one of the subgroups is 0.15, and the biomarker is both predictive and prognostic. Optimal decision rules with three different assumptions for the biomarkers (predictive, predictive and prognostic, non-informative) and subgroup prevalence are compared with a rule just based on relevance thresholds. Power is increased under the proposed decision rules when the correct biomarker assumption is made. Since the decision thresholds incorporate sample size and subgroup prevalence information, one major limitation is that knowledge about the biomarkers must be strong enough pre-trial to prespecify the required parameters.

Nesting frequentist testing procedures within a Bayesian framework, Simon and Simon proposed a group-sequential randomized adaptive enrichment trial design that uses frequentist hypothesis tests for controlling Type I error but Bayesian modeling to select treatment-sensitive subgroups and estimate effect size [17]. The primary endpoint in their models is binary, and multiple continuous biomarkers are allowed, comprising a vector of covariates for each patient. Patients are sequentially enrolled in a total of K blocks, and enrollment criteria for the next block are refined by a decision function, which is built on the block adaptive enrichment design by Simon and Simon [21]. The final analysis is based on inverse normal combination test statistics using data from the entire trial. A prior for the response rate in each arm needs to be prespecified, which is based on both the biomarker covariates and a utility function. Different utility functions can be applied according to the trial’s goal, and the one adopted here is the expected future patient outcome penalized by accrual time. Using the conditional posterior for the previous block’s information, simulations are conducted to find the optimal enrollment criteria based on the utility function. The expected treatment effect given covariates can be estimated by the posterior predictive distribution for the response rate at the end of trial. In the presented simulation study, there are two continuous biomarkers and 300 patients accrued in two or three enrollment blocks, with three logistic and three cutpoint models for the biomarker-response relationships. An unenriched design and an adaptive enrichment strategy with prespecified fixed cutpoints are compared with the proposed design. The two adaptive enrichment designs have higher power than the unenriched design to detect a treatment sensitive subgroup, and the enrichment designs have higher power when there are three versus two enrollment blocks. Compared with the fixed cutpoint enrichment method, the proposed design generally correctly identifies the treatment-sensitive subgroup while avoiding non-ideal pre-determined cutoff points for the following enrollment criteria. Though the effect size estimation is biased under the proposed design, the bias is more severe under the unenriched design.

Graf et al. proposed to optimize design decisions using utility functions from the sponsor and public health points of view in the context of a two-stage adaptive enrichment design with a continuous biomarker [22]. Similar to Simon and Simon’s method, the proposed design’s decisions are based on frequentist hypothesis tests, while the utility functions are evaluated under the Bayesian approach. In this design, patients are classified into marker positive and marker negative groups at enrollment, and decisions can be made with respect to the full population or the marker positive subgroup only. Closed testing procedures along with Hochberg tests are used to control the family wise type I error rate. Parameters called “gain”, which quantify the benefit rendered by the trial to the sponsor and society, need to be pre-specified. The utility function under the sponsor view is the sum of the gain multiplied by the probability of claiming treatment efficacy in the full population or a marker-positive group, respectively. In addition to gain and success probabilities, the public health utility function also considers the true effect sizes in subgroups, and safety risk as a penalization parameter. Prior distributions are used to model treatment effects in each subgroup to account for uncertainty, but the authors assume that only the marker negative group can be ineffective, and only point priors are used, which leads to a single probability that the treatment is effective in just the marker positive subgroup or the full population. This optimized adaptive design is compared with a non-adaptive design when the total sample sizes are the same. The adaptive design provides larger expected utility in both utility functions only when the values are intermediate in gain from treatment efficacy and the prior point probability. One limitation is that those utility functions can only compare designs with the same total sample size and the cost of running a trial is not included.

Serving as an extension of Graf et al.’s work by incorporating a term for the trial cost in utility functions, Ondra et al. derived an adaptive two-stage partial enrichment design for a normally distributed outcome with subgroup selection and optimization of the second stage sample size [23]. In a partial enrichment design, the proportion of the marker-positive subjects enrolled does not need to be aligned with the true prevalence. At interim analysis, the trial can be stopped for futility, or continued in only the marker-positive population or the full population. The final analysis is based on the weighted inverse normal function with Bonferroni correction. Utility functions used for optimization are from societal or sponsor perspectives. Expected utility is calculated by numerical integration on the joint sampling distribution of two stage-wise test statistics, with the prior distributions for the treatment effect in each subgroup. The optimal sample size for the second stage maximizes the conditional expected utility given the first stage test statistics and sample size used, and the optimal first stage sample size maximizes the utility using the solved optimal number for the second stage. The optimization function is solved recursively by dynamic programming, and the optimal design in terms of the sample size is obtained. The optimized adaptive enrichment design is compared with an optimized single- stage design for subgroup prevalence ranging from 10 to 90%, with both weak and strong predictive biomarker priors considered. Expected utilities are higher in both sponsor and societal views in the adaptive design. Also, even if the prior distribution for the effect size used in the design differs from the true distribution, the proposed adaptive design is robust in terms of expected utilities when the biomarker’s prevalence is high enough. One limitation is that the endpoint needs to be observed immediately, which might be addressed by a short-term surrogate endpoint—though to date, validated short-term endpoints are rare in oncology.

### Frequentist Approaches

Fisher et al. proposed an adaptive multi-stage enrichment design that allows sub-group selection at an interim analysis with continuous or binary outcomes [24]. Two subpopulations are predefined, and the goal is to claim treatment efficacy in one of the subpopulations or the full population. The cumulative test statistics for the subgroups and the full population are calculated at each interim analysis and compared against efficacy and non-binding futility boundaries. To control the family-wise Type I error rate (FWER), two methods for constructing efficacy boundaries are presented. One is proposed by Rosenblum et al. that spends alpha based on the covariance matrix of test statistics by populations (two subpopulations and the full population) and by interim stages [16]. Another is the alpha reallocation approach [25, 26]. The design parameters, including sample size per stage, futility boundaries, etc., are optimized to minimize the expected number enrolled or expected trial duration using simulated annealing, with constraints on power and Type I error. If the resulting design does not meet the power requirement, the total sample size will be increased until the power requirement is met. The optimized adaptive design is compared with a single-stage design, optimized single-stage design, and a multi-stage group sequential design with O’Brien-Fleming or Pocock boundaries using actual trial data from MISTIE [27] and ADNI [28]. For the MISTIE trail, the proposed designs are optimized by the expected number enrolled, which is lower than for the optimized single-stage design and group-sequential design, but the maximum number enrolled is still lower in the simple single-stage design. In the ADNI trial, when the expected trial duration is optimized, the proposed design has a slightly shorter expected duration but a longer maximum duration than the optimized single-stage design.

Similar to the aforementioned Bayesian approaches without predefined sub-populations, Zhang et al. proposed a two-stage adaptive enrichment design that does not require predefined subgroups [29]. The primary outcome is binary, and a collection of baseline covariates, including biomarkers and demographics, is used to define a treatment-sensitive subgroup. The selection criteria are based on a prespecified function modeling the treatment effect and marker by treatment interaction using first stage data. The final treatment effect estimate is a weighted average of estimates in each stage. To minimize the resubstitution bias from using first stage data in subsequent subgroup selection and inference, four methods for estimating the treatment effect and variance for the first stage are discussed: naive approach, cross-validation, nonparametric bootstrap, and parametric bootstrap. To compare those estimation methods, ECHO [30] and THRIVE [31] trial data are used for the simulation with a total sample size of 1000. The first stage has 250, 500 or 750 subjects, and the function used to simulate outcomes is the logistic regression model. The results show that the bootstrap method is more favorable than both the naive estimate (which has a large empirical bias) and the cross-validation method (which is overly conservative). The weight for each stage and first stage sample size need to be selected carefully to reach a small root mean squared error (RMSE) and close-to-nominal one-sided coverage. Though a trial can stop due to inability to recruit to a subset resulting from restricted enrollment, the proposed method does not include an early stopping rule for futility or efficacy.

In order to reduce sample size while assessing the treatment effect in the full population, Matsui and Crowley proposed a two-stage subgroup-focused sequential design for time-to-event outcomes, which could extend to multiple stages [32]. In this design, patients are classified into two subgroups by a dichotomized predictive marker, with the assumption that the experimental treatment is more efficacious in the marker-positive subgroup. The trial can proceed to the second stage with one of the subgroups, or the full population, but treatment efficacy is only tested in the marker-positive group or the full population at the final analysis. Choices of testing procedures are fixed-sequence and split-alpha. At the interim analysis, a superiority boundary for the marker-positive subgroup and a futility boundary for the marker-negative subgroup are constructed. The superiority boundary is calculated to control the study-wide alpha level, while the futility boundary is based on a Bayesian posterior probability of efficacy with a non-informative prior. The required sample sizes for each subgroup are calculated separately, and the hazard ratio for the marker-positive subgroup is recommended to be 0.05–0.70 under this application. The proposed design is compared with a traditional all-comers design, an enriched design with only marker-positive subjects, a two-stage enriched design, and a traditional marker-stratified design. Different scenarios are considered including those with no treatment effect, constant treatment effect in both groups with hazard ratio (HR) = 0.75, a nearly qualitative interaction with HRs = 0.65 and 1, and a quantitative interaction with HRs = 0.7 and 0.8. The marker prevalence is set to 0.4, and the accrual rate is 200 patients per year. When using the split-alpha test, the proposed design has greater than 80% power to reject any null hypothesis in the alternative cases, but the traditional marker-stratified design also provides enough power under all cases. The number screened and the number randomized are reduced for the proposed design compared to the traditional marker stratified design, but the reduction is only moderate.

To determine whether the full population or only the biomarker-positive subgroup benefit more from the experimental treatment, Uozumi and Hamada proposed a two-stage adaptive population selection design for a time-to-event outcome, an extension of methods from Brannath et al. and Jenkins et al. [20, 33, 34]. The main extension is that the decision-making strategy at the interim analysis incorporates both progression-free survival (PSF) and overall survival (OS) information. Also, OS is decomposed into time-to-progression (TTP) and post-progression survival (PPS) when tumor progression has occurred, to account for the correlation between OS and PFS. The combination test approach is used for the final analysis based on Simes’ procedure [35]. The hypothesis rejection rule for each population is a weighted inverse normal combination function with prespecified weights based on the expected number of OS events in each stage. At the interim analysis, a statistical model from Fleischer et al. under the semi-competing risks framework is applied to account for the correlation between OS and PFS [36, 37]. The interim decision rule uses the predictive power approach in each population, extending Brannath et al.’s method from single endpoint to multiple endpoints with a higher weight on PFS data due to its rapid observation. In the simulation, a dichotomized biomarker is used with a 50% prevalence. Four scenarios are considered, where hazard ratios in the marker-positive subgroup are always 0.5 and are higher in the marker-negative subgroup. For simplicity, the HR is the same for TTP, PPS, and death. FWER is controlled for all cases, but it is a little too conservative when the treatment is effective. The proposed design has a higher probability of identifying the treatment-sensitive population at the interim analysis, particularly when the PPS effect is large, those probabilities are similar between using OS or PFS alone or the combined endpoints when the PFS effect is small. One limitation of this design is that sample size calculations are not considered.

Instead of a single primary endpoint, Sinha et al. suggested a two-stage Phase III design with population enrichment for two binary co-primary endpoints, which is an extension of Magnusson and Turnbull’s work with co-primary endpoints [15, 38]. The two binary endpoints are assumed to be independent, and the efficacy goal should be reached in both endpoints. With two distinct predefined subgroups, a set of decision rules stops the non-responsive subgroups using efficient score statistics. The futility and efficacy boundary values, which do not depend on the marker prevalence, are the same for both endpoints due to independence. The lower and upper stopping boundaries are calculated by alpha spending functions, and FWER is strongly controlled. Simulations were conducted assuming biomarker prevalences of 0.25 or 0.75 and weighted subgroup effect sizes of 0, 1, and 2 as the means of efficient score statistics under normal distribution. The results show that the proposed design can reduce false-negative results for heterogeneous treatment effects between subgroups. The authors state the possibility of extending the design to a bivariate continuous outcome, while an extension to bivariate survival would be more challenging.

## Published Comparisons and Examination of Features

Kimani, Todd, and Stallard derived a uniformly minimum variance unbiased point estimator (UMVUE) of treatment effect in adaptive two-arm, two-stage enrichment design with a binary biomarker [39]. Based on the Rao-Blackwell theorem, UMVUE for the treatment effect conditional on the selected subgroup is derived with and without prior information on maker prevalence. The proposed estimator is compared with the naive estimator, which is biased but with a lower mean squared error (MSE) when prevalence is known. The estimator is robust, with and without prior information on marker prevalence.

Kimani et al. developed estimators for a two-stage adaptive enrichment design with a normally distributed outcome [40]. A predictive continuous biomarker is used to partition the full population into a prespecified number of subgroups, and the cutoff values are determined at the interim analyses based on stage I observations. To estimate the treatment effect after enrichment for the selected subgroup, a naive estimator, uniformly minimum variance conditional unbiased estimator (UMVCUE), unbiased estimator, single- iteration and multiple-iteration biased-adjusted estimators, and two shrinkage estimators are derived and compared. Though no estimator is superior in terms of bias and MSE in all scenarios, UMVUE is recommended by the authors due to its mean unbiasedness.

Tang et al. evaluated several proposed adaptive enrichment designs with a binary biomarker against the traditional group sequential design (GSD) for a time-to-event outcome [41]. Type I error is controlled, and the subpopulation is selected by Bayesian predictive power. Adaptive design A selects the subgroup after considering futility and efficacy stopping decision. Design B selects the subgroup when the targeted number of events are observed in full population, which can be earlier than the interim analysis. Design C selects the subgroup only after the full population has reached a futility rule. Design D proceeds with the subgroup or full population by checking the treatment effect in the complementary subgroup, proposed by Wang et al. [42]. When an enhanced treatment effect exists in the subpopulation, all of these adaptive designs could improve study power compared to GSD. Furthermore, Design C generally provides higher power across all scenarios among all the adaptive designs.

Benner and Kieser explored how the timing of interim analyses would affect power in adaptive enrichment designs with a fixed total sample size for a continuous outcome and binary marker [43]. Two subgroup selection rules are considered: the estimated treatment effect, or the estimated treatment effect difference between the subgroup and the full population (as opposed to the complement of the subgroup). When using the first selection rule, early timing increases power when the marker prevalence and marker cutoff values are low. However, the interim analysis timing’s impact on power is small when marker prevalence is high. If absolute treatment effect is used instead, earlier timing leads to power loss in general. Power depends more on the marker threshold, prevalence, and treatment effect size when interim timing is later than when half of the total sample size have observed outcomes.

Kunzmann et al. investigated the performance of six different estimators besides maximum likelihood estimator (MLE) for a two-stage adaptive enrichment design for a continuous outcome [44]. Those estimators are empirical Bayes estimator (EBE) [45, 46], parametric bootstrap estimator [47], conditional moment estimator (CME) [48], and UMVCUE with MLE and CME as two hybrid estimators [49]. The hybrid UMVCUE and CME estimator could reduce the bias across all considered scenarios, which the authors recommend, though with the cost of larger RMSE.

## Conclusions and Future Needs in Adaptive Enrichment Trial Designs

In this review article, we have given an overview of traditional enrichment and adaptive enrichment designs, outlined their limitations, and described recent extensions and modifications to adaptive enrichment design strategies. Both Bayesian and frequentist perspectives in handling statistical issues of these designs were discussed in detail, along with important considerations for design parameters.

Although the adaptive enrichment designs we have reviewed contain theoretical benefits such as early subgroup identification and early decision-making resulting in sample size reduction, we caution that selection and implementation of any of these designs requires acceptance of substantial additional trial complexity, and special consideration of the disease setting, endpoints, and markers at hand. For any of these trial designs to possibly have advantages over a simple randomized design followed by retrospective biomarker-focused analyses, the following should be true: the primary endpoint should be quickly observable relative to the pace of accrual; a sufficiently large sample size to detect moderately-sized subgroup effects of clinical interest must be achievable in a reasonable time frame, and the experimental treatment under study must have sufficiently strong preliminary evidence (e.g., from earlier phase studies) of a mechanism of action related to the candidate biomarker(s). If any of these criteria are not met, one runs the serious risk of conducting a study that is far less efficient than a standard design that is not biomarker-driven. In considering use of any design considered here, a trial biostatistician should meet with trial investigators and stakeholders to discuss the assumptions and requirements of different design options. The statistician should also prospectively understand and quantify the impact of any potential deviations from these assumptions while still in the trial planning stage (e.g., by using simulation studies).

Each of the designs we discussed also have associated pros and cons, and are more suitable for application in different settings. To guide selection of a particular design for a particular context, we summarize design attributes (e.g., applicable primary endpoint types, number of biomarkers, decision rules, and other structural differences) as well as pros and cons in Table 1. For example, if there is no predefined biomarker subgroup and predictive biomarker discovery is required, Xu et al. and Zhang et al.’s proposed designs could be considered [10, 29]. Where Bayesian methods for estimation and interim decision-making using utility functions are desired but where final frequentist hypothesis testing is necessary, e.g., for regulatory purposes, the designs by Simon and Simon, Graf et al., or Ondra et al. may be appropriate [17, 22, 23]. Where strong control of Type I error rate is required (e.g., in a later-phase application), designs by Matsui and Crowley, Fisher et al., and Uozumi and Hamada may be referenced [24, 32, 34].

**Table 1.** Summary Table for Recent Developments and Extensions in Adaptive Enrichment Trial Designs

Adaptive Enrichment Design Method	Type of Endpoints	Article	Design or Model Name or Title	Biomarker Types (Number Allowed^a^)	Pros	Cons
Bayesian	Time-to-event outcome	Park et al. [14]	Group sequential adaptive enrichment design	Continuous (50)	• An early response indicator is incorporated • Biomarkers selection	• Biomarkers need to be independent (though selection algorithm can be extended to deal with correlated biomarkers)
		Ohwada and Morita [18]	Bayesian adaptive patient enrollment restriction (BAPER)	Continuous (1)	• Subgroup identification	• Monotonicity in biomarker and treatment effect interaction • No prognostic biomarker
	Binary outcome	Krisam and Kieser [19]	Optimal Decision Rules for Biomarker-Based Subgroup Selection for a Targeted Therapy in Oncology	Binary (1)	• Biomarker can be predictive and/or prognostic • Tables of optimal decision threshold of various sample size by subgroup prevalence are provided	• Monotonicity in biomarker and treatment effect interaction • One interim analysis • Required prior knowledge to prespecify decision thresholds
		Simon and Simon [17]	Using Bayesian modeling in frequentist adaptive enrichment designs	Continuous (2)	• Combine Bayesian methods and Frequentist hypothesis test • Different utility functions can be used • Biomarker can be predictive and/or prognostic	• Monotonicity in biomarker and treatment effect interaction • Small bias in treatment effect estimations
	Continuous outcome	Graf et al. [22]	Adaptive designs for subpopulation analysis optimizing utility functions	Binary (1)	• Combine Bayesian methods and Frequentist hypothesis test • Utility functions can be extended • Control FWER	• Utility functions can only compare across designs with the same total sample size • Monotonicity in biomarker and treatment effect interaction
		Ondra et al. [23]	Optimized adaptive enrichment designs	Binary (1)	• Combine Bayesian methods and Frequentist hypothesis test • Utility functions can represent sponsor’s view or societal view • Control FWER	• Monotonicity in biomarker and treatment effect interaction
	Binary/Categorical/Continuous outcome	Xu et al. [10]	Adaptive subgroup-identification enrichment design (ASIED)	Continuous/Binary/Categorical/Ordinal (4)	• Biomarkers selection • Alternative subgroup identification model can be used • Small sample size allowed • Minimal and target levels of treatment effect can be specified	• Up to two interim analyses allowed
Frequentist	Time-to-event outcome	Matsui and Crowley [32]	Subgroup-focused marker-stratified sequential design	Binary (1)	• Control study-wise Type I error • Futility rules are based on Bayesian posterior probability • Reduce number of patients enrolled and study duration	• Monotonicity in biomarker and treatment effect interaction • One interim analysis • Small increase in power compared to traditional marker stratified design
		Uozumi and Hamada [34]	Interim decision-making strategies in adaptive designs for population selection using time-to-event endpoints	Binary (1)	• Interim decision rules incorporated both PSF and OS • Control FWER	• Monotonicity in biomarker and treatment effect interaction • One interim analysis • No sample size estimation method provided
	Binary outcome	Zhang et al. [29]	Treatment evaluation for a data-driven subgroup in adaptive enrichment designs of clinical trials	Continuous/Binary/Categorical (6)	• Reduce resubstitution bias • Subgroup identification	• No early stopping rules for treatment effect • One interim analysis
		Sinha et al. [38]	Group-sequential adaptive enrichment design with two binary co-primary endpoints	Binary (1)	• Two binary co-primary endpoints • Control FWER	• Independent co-primary endpoints • One interim analysis
	Binary/Continuous outcome	Fisher et al. [24]	Stochastic optimization of adaptive enrichment designs for two subpopulations	Binary (1)	• Control FWER and meet power requirement • Expected sample size or trial duration can be minimized	• Computationally intensive

^a^If maximum number of allowed biomarkers was not specified, the number of biomarkers used in simulations will be stated

Overall, adaptive enrichment trial designs tend to increase study efficiency while minimizing subsequent study participation among patients showing a low likelihood of benefit based on early trial results [21]. Biomarker-driven designs that reliably identify or validate predictive biomarker relationships and their thresholds with sufficient power to achieve phase II or III objectives continue to be of interest and warrant further development. Designs that make better use of truly continuous (versus dichotomous) marker-efficacy relationships are essential for future research.

## Data Availability

No datasets were generated or analysed during the current study.
